# Effect of humeral rotation on rotator cuff strain, loading and kinematics: an in vitro study

**DOI:** 10.1186/s12938-025-01406-4

**Published:** 2025-06-20

**Authors:** Inês Santos, Lieselotte Pichler, Matthias F. Pietschmann, Mark Tauber, Peter E. Müller

**Affiliations:** 1 Musculoskeletal University Center Munich (MUM), University Hospital, LMU Munich, Munich, Germany; 2 OrthoPaxis Oberhaching, Oberhaching, Germany; 3 ATOS Clinic Munich, Munich, Germany; 4 Musculoskeletal University Center Munich (MUM), Fraunhoferstr. 20, 82152 Planegg, Germany

**Keywords:** Supraspinatus tear, Rotator cuff, Strain, Shoulder kinematics, Humeral head rotation

## Abstract

**Background:**

Despite its main function as abductor, the role of the supraspinatus as stabilizer and rotator cannot be neglected. A supraspinatus tear may not only influence humeral head rotation during abduction but also the strength and loading of the acting (intact) rotator cuff muscles. The purpose of this study was to investigate the effect of constrained humeral rotation and elevation on rotator cuff loading, strain and kinematics with intact and torn cuff conditions.

**Methods:**

Active humeral elevation until 30° was simulated in twelve fresh-frozen cadaver shoulders with free humeral rotation and blocked humeral rotation. The loading protocol was applied to the intact rotator cuff, and after a 50% and 100% wide (full-thickness) crescent-shaped (*n* = 6) and reverse L-shaped (*n* = 6) tears were created in the supraspinatus tendon.

**Results:**

Constrained humeral rotation led to an increase in supraspinatus loading force and maximum supraspinatus strain for both tear shapes. Range of motion was significantly reduced in 7 of the 12 specimens due to blocked humeral rotation. In the 100% wide reverse L-shaped tear group, constrained rotation led to an anterior translation of humeral head, in contrast to the posterior translation observed with free rotation.

**Conclusions:**

Blocking humeral head rotation leads to an increase in supraspinatus and infraspinatus strains. According to its function as external rotator of the shoulder, the strain in the infraspinatus was higher at the beginning of abduction. However, small rotator cuff tears might not biomechanically result in increased humeral rotation, possibly because the load on the infraspinatus is compensated by the subscapularis.

**Level of evidence:**

Basic Science Study; Biomechanics.

## Introduction

Rotator cuff tear (RCT) arthropathy is a common shoulder disorder associated with pain and limited range of motion, and its prevalence in the general population increases with age [23, 26]. Factors such as genetic predisposition, extrinsic impingement and biomechanical imbalance from structures surrounding the rotator cuff, as well as tendon degeneration have been highlighted as etiological factors [10, 13, 14]. Glenohumeral osteoarthritis has been shown to cause progressive fatty infiltration of the rotator cuff, potentially contributing to tear formation and progression [21]. Previous studies have demonstrated that the rotator cuff generates the necessary torque for rotation of the humerus and compression of the humeral head into the glenoid cavity [1, 7, 17]. However, the presence of a RCT results in unbalanced dynamic stabilization of the glenohumeral joint, leading to loss of active elevation and rotation as a compensatory mechanism to reduce pain [20]. This pain is believed to be a parallel process likely related to excessive strain or wear on the remaining cuff [8]. The supraspinatus (SSP) tendon is a common site of injury and it is unclear how the loss of its function as abductor and rotator influences the strength and loading distribution on the acting, still intact, rotator cuff muscles.

This study aimed to evaluate rotator cuff loading, strain and glenohumeral kinematics in mid-stage glenohumeral osteoarthritis by simulating a torn SSP and restricted humeral rotation, as observed following osteophyte formation in the osteoarthritic glenohumeral joint. Two commonly occurring tear shapes—crescent (CS) and reverse L-shaped (rLS)—of different sizes (50% and 100% width) were investigated. Together with the L-shaped tears, they account for 70% of posterosuperior rotator cuff tears [9]. However, the effect of the L-shaped tear was not assessed in this study. We hypothesized that: (1) constrained humeral rotation will lead to reduced range of motion; (2) humeral head translation will differ between tear shapes due to constrained and free rotation; (3) constrained rotation will lead to an increase in SSP loading force and (4) maximum strain.

## Results

An overview of the specimen characteristics, generated tear shape and the AP and ML dimensions of the SSP tears created in each specimen are presented in Table 1. The SSP tendon of specimen nr. 10 ruptured during the last test series (100% wide tear with blocked humeral rotation) and, therefore, the values corresponding to this test were excluded from the results analysis.

**Table 1:** Overview of specimen characteristics, generated tear shape and supraspinatus tear dimensions

Specimen	Age	Gender	Side	Tear shape	AP tear size [mm]		ML tear size [mm]			
					50% wide	100% wide	50% wide CS	100% wide CS	50% wide rLS	100% wide rLS
1	74	F	L	CS	8.6	17.2	4.3	8.6	–	–
2	66	M	R	CS	6.9	13.7	3.5	6.9	–	–
3	70	F	R	CS	5.8	11.7	2.9	5.8	–	–
4	79	M	R	CS	8.3	16.6	4.2	8.3	–	–
5	82	M	L	CS	6.7	13.4	3.4	6.7	–	–
6	82	M	R	CS	7.2	14.3	3.6	7.2	–	–
7	66	M	L	rLS	7.5	15.0	–	–	7.5	15.0
8	74	M	R	rLS	6.7	13.4	–	–	6.7	13.4
9	78	F	R	rLS	8.9	17.8	–	–	8.9	17.8
10	70	F	L	rLS	4.5	8.9	–	–	4.5	8.9
11	84	M	R	rLS	7.3	14.7	–	–	7.3	14.7
12	36	M	R	rLS	7.6	15.1	–	–	7.6	15.1
Mean ± SD	72 ± 13	–	–	–	7.2 ± 1.2	14.3 ± 2.4	3.6 ± 0.5	7.3 ± 1.0	7.1 ± 1.5	14.2 ± 2.9

^a^AP: anterior–posterior; CS: crescent-shaped; F: female; L: left; M: male; ML: medial–lateral; R: right; rLS: reverse L-shaped; SD: standard deviation

### Humeral head translation

A posterior translation of the humeral head could be observed in 11 of 12 specimens with intact SSP tendon. After the 50% wide CS tear was surgically created, cranialization was observed in both test series (+R, 4 of 6 specimens; −R, all specimens). In the 50% wide rLS group, the SSP tear led to cranialization and translation of the humeral head in the posterior direction during the free humeral rotation test series (+R, 4 of 6 specimens). No trend in translation in the AP direction could be recognized after blocking humeral rotation in the rLS tear group.

Tear extension to 100% width of the tendon width led to cranialization in both tear shape groups (CS, 4 of 6 specimens; rLS, 4 of 5 specimens) for both test series. A translation of the humeral head in the anterior direction could be observed in the CS tear group with free and blocked humeral rotation (+R, 4 of 6 specimens; −R, 5 of 6 specimens). A difference between free and blocked rotation could be seen in the rLS tear group: a translation of the CoR in the posterior direction occurred with free humeral rotation (4 of 5 specimens), while with constrained rotation the humerus moved anteriorly (3 of 5 specimens). The differences observed in translation in the AP and superior–inferior directions between the +R and −R test series were not statistically significant (*P* = 0.06 and *P* = 0.37, respectively).

### Range of motion

In the –R test series, internal rotation could successfully be reduced (*P* < 0.0007). The trend of humeral rotation between the various tendon conditions (intact, 50% wide and 100% wide tear) was not the same for the test series +R and –R (Table 2). In the –R test series only 3 of 12 specimens with an intact SSP, 5 of 12 specimens in the 50% wide and 5 of 12 specimens in the 100% wide tear groups reached a maximum abduction angle of 30°. In the test series with free rotation this was the case for 11 of 12 specimens. In the CS tear group, only 3 specimens with a 50% wide tear and 4 specimens with a 100% wide tear reached a minimum abduction of 15°, whereas in the rLS group, this was observed in 4 specimens.

**Table 2:** Maximum abduction angle measured for each specimen in the test series with blocked rotation

Specimen	Maximum abduction angle [°] with constrained humeral rotation (−R)					
	CS tear group			rLS tear group		
	Intact	50% wide	100% wide	Intact	50% wide	100%wide
1	30.0	30.0	30.0	–	–	–
2	15.0	14.0	15.5	–	–	–
3	26.0	30.0	30.0	–	–	–
4	9.0	9.5	8.0	–	–	–
5	16.5	18.0	22.0	–	–	–
6	5.5	8.0	11.5	–	–	–
7	–	–	–	30.0	30.0	30.0
8	–	–	–	30.0	30.0	30.0
9	–	–	–	7.5	9.5	7.0
10	–	–	–	17.0	3.5	–^*^
11	–	–	–	27.0	30.0	30.0
12	–	–	–	8.5	19.0	23.5
Mean ± SD	17 ± 9.5	18.3 ± 9.8	19.5 ± 9.4	20 ± 10.5	20.3 ± 11.7	24.1 ± 1

CS: crescent-shaped; rLS: reverse L-shaped; SD: standard deviation

^*^Specimen 10 was excluded from the results analysis due to extensive SSP damage during the test series with constrained rotation

### Maximum SSP loading force

**Fig. 1:**
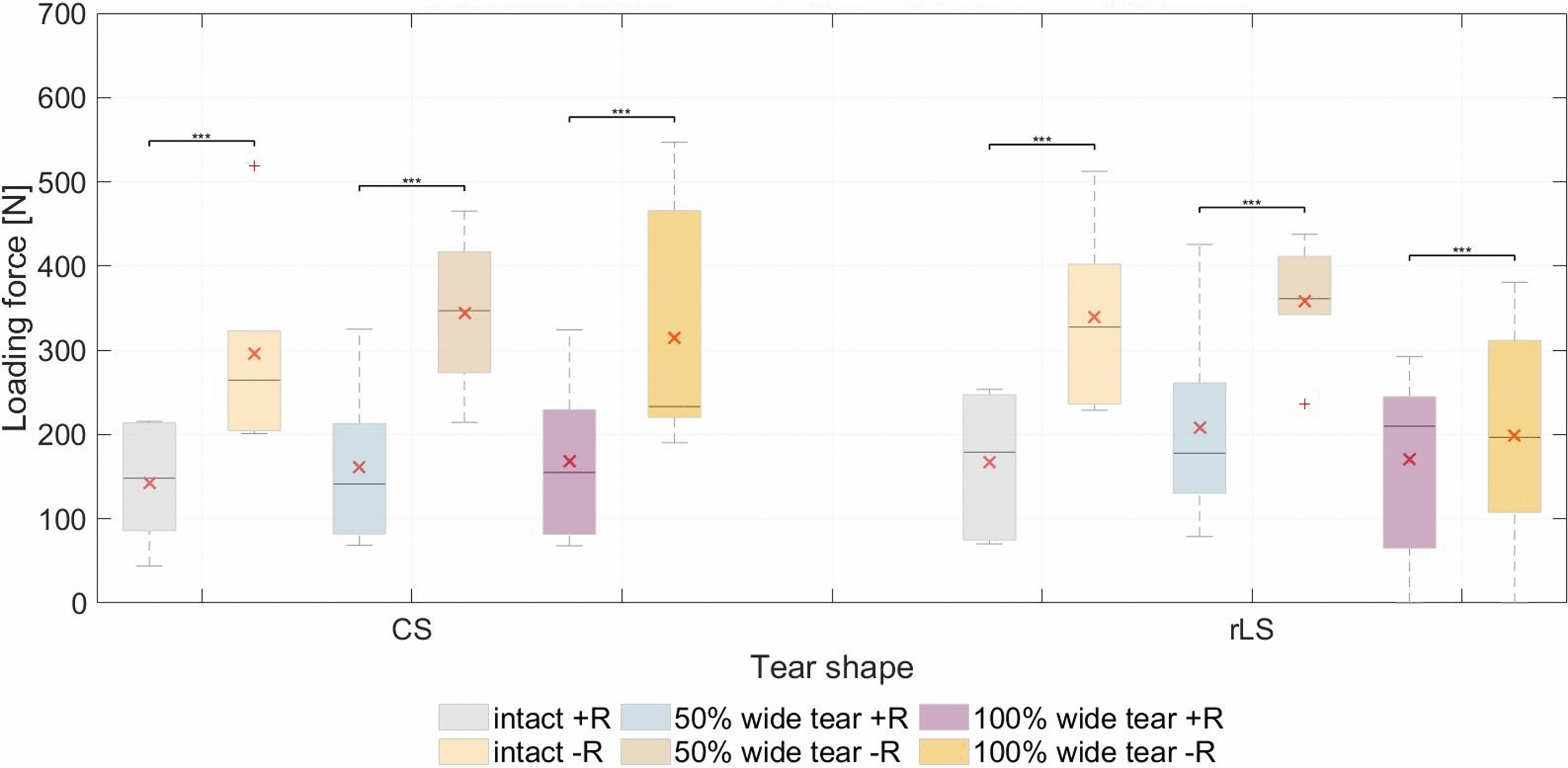
Maximum loading force measured at the supraspinatus tendon. The mean values (in degrees) are indicated (×). ***P < 0.0001. CS, crescent-shaped tear; rLS, reverse L-shaped tear; +R, free humeral rotation; −R, constrained humeral rotation

Blocking humeral rotation led to an increase in the SSP loading force necessary to abduct the humerus (Fig. 1, Table 3). More specifically, in comparison to the free rotation (+ R) test series, the SSP loading force in the CS group was for 5 of 6 specimens 187.65 ± 118.42% higher with an intact SSP. After creating a 50% wide tear, the SSP loading force increased 187.43 ± 163.71% (all specimens) and after tear extension to 100% of the SSP width, it increased 147.13 ± 75.15% in 5 of 6 specimens. For the rLS group, the SSP loading force was 141.95 ± 112.50% higher than in the free rotation (+ R) test series with an intact SSP (all specimens). Creation of a 50% wide tear led to an increase in SSP loading force of 157.65 ± 152.95% in 5 of 6 specimens and tear extension to 100% width resulted in an increase of 39.04 ± 20.75% in 4 of 5 specimens.

**Table 3:** Maximum supraspinatus loading force measured for each specimen in the test series with free and blocked rotation

Specimen	Maximum SSP loading force [N]											
	+R						−R					
	CS tear group			rLS tear group			CS tear group			rLS tear group		
	Intact	50% wide	100% wide	Intact	50% wide	100% wide	Intact	50% wide	100% wide	Intact	50% wide	100%wide
1	213.9 ± 4.3	212.8 ± 2.5	227.7 ± 5.6	–	–	–	209.4 ± 4.0	214.4 ± 1.1	227.4 ± 6.2	–	–	–
2	96.0 ± 15.4	82.8 ± 2.2	82.2 ± 0.9	–	–	–	323.1 ± 64.1	273.5 ± 122.3	190.2 ± 261.3	–	–	–
3	215.6 ± 8.4	325.1 ± 4.9	324.2 ± 18.8	–	–	–	518.3 ± 12.0	417.0 ± 81.6	465.8 ± 185.9	–	–	–
4	85.7 ± 2.5	81.9 ± 1.0	67.9 ± 3.0	–	–	–	201.1 ± 194.9	340.8 ± 23.7	239.0 ± 139.7	–	–	–
5	200.4 ± 13.7	199.9 ± 1.5	229.5 ± 14.8	–	–	–	319.7 ± 74.6	465.1 ± 40.7	546.8 ± 7.8	–	–	–
6	43.8 ± 15.3	68.4 ± 14.0	81.5 ± 2.9	–	–	–	204.5 ± 113.3	353.2 ± 1.0	220.3 ± 151.0	–	–	–
7	–	–	–	193.6 ± 6.8	201.6 ± 3.0	203.7 ± 0.3	–	–	–	228.8 ± 5.0	235.7 ± 0.4	237.6 ± 0.9
8	–	–	–	253.5 ± 0.2	261.2 ± 11.3	245.1 ± 0.6	–	–	–	351.2 ± 50.7	342.2 ± 28.3	155.5 ± 214.2
9	–	–	–	74.7 ± 16.3	78.9 ± 2.6	65.1 ± 2.8	–	–	–	303.8 ± 15.0	380.2 ± 27.1	107.6 ± 3.9
10	–	–	–	164.4 ± 4.0	130.3 ± 3.1	–^*^	–	–	–	402.3 ± 35.7	437.7 ± 3.7	–^*^
11	–	–	–	247.3 ± 4.1	425.6 ± 82.2	292.7 ± 0.2	–	–	–	512.3 ± 40.8	411.5 ± 66.8	380.6 ± 5.2
12	–	–	–	70.2 ± 0.2	154.0 ± 4.6	216.1 ± 6.2	–	–	–	236.1 ± 231.4	342.3 ± 236.0	311.8 ± 91.8
Median	148.2	141.3	155.0	179.0	177.8	209.9	264.5	347.0	233.2	327.5	361.3	196.6
Mean ± SD	142.6 ± 76.0	161.8 ± 102.1	168.8 ± 106.4	167.3 ± 80.7	208.6 ± 123.2	170.5 ± 113.0	296.0 ± 122.9	344.0 ± 91.5	314.9 ± 151.3	339.1 ± 107.9	358.3 ± 70.9	198.9 ± 139.3

CS: crescent-shaped; rLS: reverse L-shaped; SD: standard deviation; SSP: supraspinatus; + R: free humeral rotation; −R: constrained humeral rotation

^*^Specimen 10 was excluded from the results analysis due to extensive SSP damage during the test series with constrained rotation

One specimen (specimen 2) in the CS group and two (specimens 7 and 9) in the rLS group showed the same SSP loading trend between the various conditions of the SSP tendon (intact, 50% wide and 100% wide tear) in both test series (+ R and –R). The SSP loading force differed significantly between the + R and −R test series (*P* = 0.0001).

### Strain at anterior and posterior borders of tear in relation to mean SSP tendon strain

After creating the 50% wide tear, maximum strain was measured at approximately the same range of abduction (± 5°) in both test series, +R and −R, in 2 of 3 specimens of the CS tear group. After tear extension to 100% width, maximum strain continued to be measured within the same range of abduction for both test series in 2 of 4 specimens. Regarding the location of maximum strain at the borders, a similar trend was observed in both test series (+R and −R) for the CS tear group. However, no general trend in location could be observed for all specimens. In the rLS tear group, no clear trend was observed in maximum SSP strain at the tear borders for both tear size and humeral rotation test series.

### Strain in SSP and ISP adjacent to footprint

**Fig. 2:**
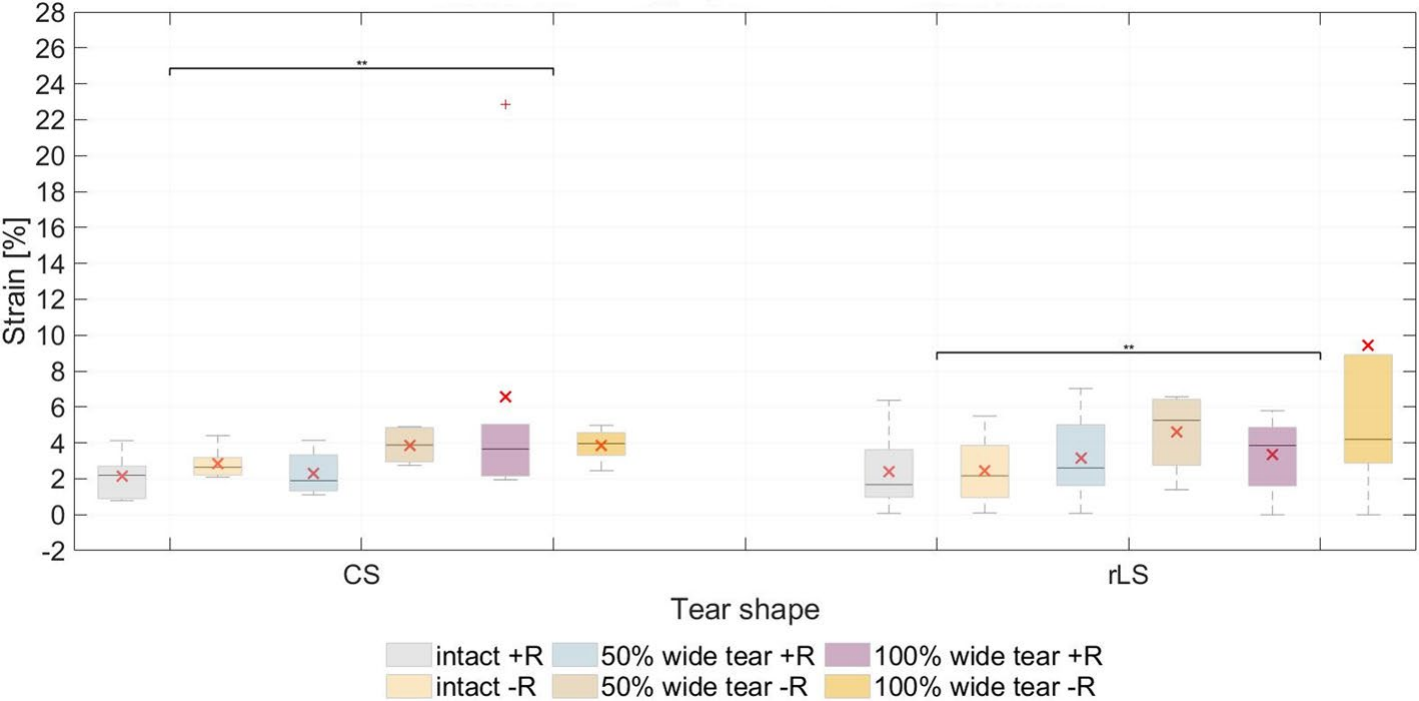
Maximum supraspinatus strain measured in the test series with free and blocked rotation. The mean values (in degrees) are indicated (×). CS, crescent-shaped tear; rLS, reverse L-shaped tear; +R, free humeral rotation; −R, constrained humeral rotation

Blocking humeral rotation led to an increase in average maximum SSP strain in both intact and torn conditions (except for the 100% wide CS tear group) (Fig. 2, Table 4). This increase was higher in the 50% wide CS tear group (205,3%) and 50% wide rLS tear group (203,9%). The difference between the intact SSP and the 100% wide tear was significant for both tear shapes (*P* = 0.0088). Between the test series (+R and −R), however, no significant effect could be identified.

**Table 4:** Maximum SSP strain measured for each specimen in the test series with free and blocked rotation

Specimen	Maximum SSP strain [%]											
	+R						−R					
	CS tear group			rLS tear group			CS tear group			rLS tear group		
	Intact	50% wide	100% wide	Intact	50% wide	100% wide	Intact	50% wide	100% wide	Intact	50% wide	100%wide
1	2.4 ± 0.4	2.1 ± 0.2	4.9 ± 0.4	–	–	–	2.1 ± 0.4	4.2 ± 1.3	5.0 ± 1.2	–	–	–
2	0.9 ± 0.0	1.3 ± 0.2	1.9 ± 0.5	–	–	–	2.3 ± 0.2	2.7 ± 0.8	2.5 ± 1.6	–	–	–
3	4.1 ± 0.1	4.1 ± 0.2	22.9 ± 14.4	–	–	–	4.4 ± 0.3	4.9 ± 0.2	4.6 ± 0.2	–	–	–
4	2.7 ± 0.1	3.3 ± 0.1	5.0 ± 1.4	–	–	–	3.2 ± 1.6	4.9 ± 0.0	4.3 ± 2.2	–	–	–
5	2.0 ± 0.3	1.7 ± 0.2	2.2 ± 0.1	–	–	–	3.0 ± 1.1	2.9 ± 0.2	3.6 ± 0.2	–	–	–
6	0.8 ± 0.4	1.1 ± 0.1	2.4 ± 0.2	–	–	–	2.2 ± 0.2	3.6 ± 0.7	3.3 ± 1.4	–	–	–
7	–	–	–	1.0 ± 0.0	3.0 ± 0.1	2.8 ± 0.5	–	–	–	1.0 ± 0.3	2.8 ± 0.4	2.9 ± 0.7
8	–	–	–	6.4 ± 0.1	5.0 ± 0.7	4.9 ± 0.7	–	–	–	5.5 ± 0.5	6.4 ± 0.6	36.3 ± 44.8
9	–	–	–	1.5 ± 0.3	2.2 ± 0.2	4.9 ± 0.6	–	–	–	3.9 ± 0.5	5.5 ± 0.2	8.9 ± 6.0
10	–	–	–	1.8 ± 0.2	1.6 ± 0.0	–^*^	–	–	–	2.4 ± 0.3	5.0 ± 1.0	–^*^
11	–	–	–	3.6 ± 0.2	7.0 ± 2.9	5.8 ± 1.5	–	–	–	1.9 ± 2.7	6.6 ± 1.4	3.2 ± 4.0
12	–	–	–	0.1 ± 0.1	0.1 ± 0.1	1.6 ± 2.2	–	–	–	0.1 ± 0.0	1.4 ± 1.1	5.2 ± 0.2
Median	2.2	1.9	3.7	1.7	2.6	3.9	2.6	3.9	4.0	2.2	5.3	4.2
Mean ± SD	2.1 ± 1.2	2.3 ± 1.2	6.6 ± 8.1	2.4 ± 2.3	3.2 ± 2.5	3.3 ± 2.2	2.9 ± 0.9	3.9 ± 0.9	3.9 ± 0.9	2.5 ± 2.0	4.6 ± 2.1	9.4 ± 13.5

CS: crescent-shaped; rLS: reverse L-shaped; SD: standard deviation; SSP: supraspinatus; +R: free humeral rotation; −R: constrained humeral rotation

.^*^Specimen 10 was excluded from the results analysis due to extensive SSP damage during the test series with constrained rotation

**Fig. 3:**
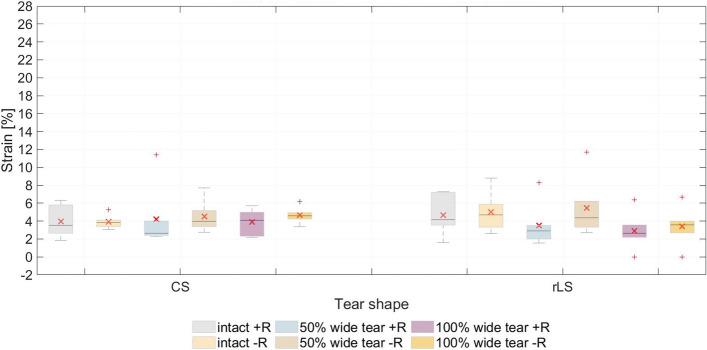
Maximum infraspinatus strain measured in the test series with free and blocked rotation. Mean values (in degrees) are indicated (×). CS, crescent-shaped tear; rLS, reverse L-shaped tear; +R, free humeral rotation; −R, constrained humeral rotation

Strain in the ISP was higher than that in the SSP tendon, during the simulated abduction range, for the 50% wide group (CS tear, *n* = 5; rLS tear, *n* = 4) and for the 100% wide CS tear group (*n* = 5). The difference between ISP and SSP was not significant either between tear shapes or between tear widths in the analyzed range of abduction. Blocking humeral rotation resulted in an increase in ISP strain (Fig. 3, Table 5).

**Table 5:** Maximum infraspinatus strain measured for each specimen in the test series with free and blocked rotation

Specimen	Maximum ISP strain [%]											
	+R						−R					
	CS tear group			rLS tear group			CS tear group			rLS tear group		
	Intact	50% wide	100% wide	Intact	50% wide	100% wide	Intact	50% wide	100% wide	Intact	50% wide	100%wide
1	6.3 ± 1.8	11.4 ± 4.0	5.7 ± 0.4	–	–	–	4.1 ± 0.4	3.9 ± 0.8	4.5 ± 0.2	–	–	–
2	2.6 ± 0.1	2.7 ± 0.1	2.2 ± 0.1	–	–	–	3.1 ± 0.0	2.7 ± 0.0	3.4 ± 0.9	–	–	–
3	1.8 ± 0.2	2.3 ± 0.1	5.0 ± 3.2	–	–	–	3.9 ± 2.0	5.2 ± 0.0	4.7 ± 1.8	–	–	–
4	2.7 ± 0.1	2.6 ± 0.2	2.3 ± 0.1	–	–	–	3.4 ± 0.2	3.4 ± 0.2	4.2 ± 0.6	–	–	–
5	5.8 ± 1.0	2.4 ± 0.0	3.4 ± 0.4	–	–	–	5.3 ± 1.0	7.7 ± 0.3	6.2 ± 2.3	–	–	–
6	4.4 ± 0.7	4.0 ± 1.0	4.7 ± 0.3	–	–	–	4.1 ± 0.4	3.9 ± 0.8	4.5 ± 0.2	–	–	–
7	–	–	–	3.5 ± 0.6	2.6 ± 0.0	3.6 ± 0.7	–	–	–	3.3 ± 0.6	2.7 ± 0.0	3.2 ± 0.0
8	–	–	–	7.2 ± 2.6	8.3 ± 0.5	6.3 ± 0.3	–	–	–	8.8 ± 2.3	11.7 ± 0.6	4.0 ± 3.1
9	–	–	–	7.3 ± 5.4	1.5 ± 0.0	2.2 ± 1.0	–	–	–	5.9 ± 0.9	3.9 ± 0.6	6.7 ± 4.9
10	–	–	–	4.0 ± 0.5	3.5 ± 0.1	–^*^	–	–	–	5.4 ± 0.6	6.2 ± 0.5	–^*^
11	–	–	–	4.3 ± 0.6	3.2 ± 0.1	3.0 ± 0.3	–	–	–	4.0 ± 0.0	3.3 ± 0.3	4.0 ± 1.0
12	–	–	–	1.6 ± 0.1	2.0 ± 0.3	2.2 ± 0.0	–	–	–	2.6 ± 1.7	4.8 ± 1.0	2.7 ± 0.2
Median	3.5	2.6	4.1	4.2	2.9	2.6	3.9	4.0	4.6	4.7	4.4	3.6
Mean ± SD	3.9 ± 1.8	4.2 ± 3.6	3.9 ± 1.5	4.7 ± 2.2	3.5 ± 2.4	2.9 ± 2.1	3.9 ± 0.8	4.5 ± 1.8	4.6 ± 0.9	5.0 ± 2.2	5.4 ± 3.3	3.4 ± 2.2

CS: crescent-shaped; ISP: infraspinatus; rLS: reverse L-shaped; SD: standard deviation; +R: free humeral rotation; −R: constrained humeral rotation

^*^Specimen 10 was excluded from the results analysis due to extensive SSP damage during the test series with constrained rotation

## Discussion

The aim of this study was to simulate a worst-case scenario for full-thickness SSP tear by simulating the loss of active elevation and rotation in abduction to 30° in the scapular plane and investigate its effect on rotator cuff load, kinematics and strain of the SSP and ISP tendons. Additionally, the possible influence of different SSP tear shapes and sizes combined with the aforementioned motion constraints was assessed.

### Humeral head translation

Superior migration of the humeral head could be observed for both tear shapes directly after tear generation in the free rotation test series. The wrapping of the humeral head provided by the SSP tendon is directly affected by the shape of the generated tear, thus influencing stabilization in the superior–inferior direction. Cranialization of the humeral head could expose the SSP tendon to mechanical friction under the acromion, leading to further tear progression. Constraining humeral rotation was expected to further disrupt glenohumeral stability. This effect, however, was more evident after the 100% wide tear was generated. After creation of the 100% wide tear, instability in the AP direction was observed in both tear shape groups, though with different patterns. In the 100% wide CS tear group the humeral head moved anteriorly, probably due to the preservation of the mechanically stronger anterior part of the SSP tendon and its interdigitation with the adjacent tendons [3]. In the rLS tear group the translational trend was not as clear. In the test series with free rotation, the humeral head moved posteriorly, whereas with blocked humeral rotation, it translated anteriorly. Translation in the posterior direction could be caused by the larger lateral to medial defect created at the anterior margin of the SSP, in comparison to its still intact posterior part. Hence emphasizing the influence of tear shape and location in humeral CoR position during glenohumeral abduction. When blocking humeral rotation, however, an additional disruption factor was introduced. An increase in humeral internal rotation after creating a 100% wide SSP tear has been shown in a previous study [19]. By constraining humeral rotation, the expected humeral motion (as seen in the free rotation test series) was disrupted. This shows that blocking humeral rotation directly affects translation, causing eccentric loading and contributing to further glenohumeral instability, especially in the case of a 100% wide rLS tear. In the long term, this increase in instability may result in degenerative arthritis [2, 22].

### SSP loading force

An increase in SSP loading force was expected with constrained humeral rotation due to the significant change in humeral CoR position and, subsequently, the reduced effectiveness of its superiorly directed force vector. The results for both tear shapes and sizes confirmed this hypothesis. However, the same trend between the different tendon conditions could not be observed. This might be explained by the altered force vector after tear generation. Interestingly, both on average and median peak SSP loading force decreased after tear extension to 100% width in the blocked humeral rotation test series (in contrast with the increase seen after creating the 50% wide tear). This suggests that a 50% wide SSP defect, though smaller, leads to a mechanically worse humeral head position than a larger defect. When comparing the influence of humeral rotation on the tear shape groups, a clear difference between CS and rLS tears could be observed with a 100% wide tear size, hinting to a resulting worse lever arm in the case of a CS tear. The increase in SSP loading force caused solely by the presence of a tear is a strong indicator of a possible tear progression. Combined with arthritic changes in the glenohumeral joint that could limit humeral rotation due to osteophyte formation, it may accelerate tear development by mechanically altering joint kinematics [24]. It is important to recall that while the deltoid can compensate for part of the loss of the SSP as abductor, possibly reducing the load on the damaged tendon, it cannot compensate for the loss of its stabilizer and rotator function.

### Strain distribution

With its insertion at the highest impression of the greater tubercle, the SSP not only functions as a shoulder abductor but as an external rotator as well [12, 15]. Therefore, the biomechanical behavior of the remaining rotator muscles with influence on rotation (ISP and SCP) is of interest.

The strain analysis showed that, although the strain of the ISP was higher at the beginning of the abduction, the predisposition for humeral external rotation did not increase in any of the test series. Even in the test with constrained rotation, the ISP strain did not increase excessively in comparison to the test series with free rotation. We believe, therefore, that the tendency for external rotation of the ISP, presumed due to the increased strain but not observed in this study, is compensated by the SCP. SCP strain could not be measured with our testing set-up, thus further investigation is required.

### Limitations

Some limitations have to be considered when interpreting the results of the present study. Both the specimen preparation and biomechanical setup resulted in a simplified model of the shoulder joint in which the coracoacromial ligament and physiological depressors (pectoralis major, latissimus dorsi, and teres major) are missing. This may have contributed to a superior migration of the humeral head. Tissue degeneration and remodeling caused by injury and/or repair are also absent from the current biomechanical model. Thus, changes in the mechanical properties of the rotator cuff tendons due to in vivo factors were not taken into account in the results interpretation. Finally, the high anatomic variability between specimens resulted in large standard deviations; therefore, additional studies with a larger sample size are required to achieve higher statistical significance levels.

## Conclusion

The findings of this study provide new insights into the effects of a torn SSP and limited rotation on rotator cuff loading, strain and kinematics. It was observed that constrained humeral rotation further disrupts glenohumeral stability, leading to an increase in SSP and ISP strains and reducing the effectiveness of the SSP superiorly directed force vector. The SSP loading force decreased after extending the tear to 100% of its width, suggesting that a smaller defect leads to a mechanically worse humeral head position than a larger defect. However, small rotator cuff tears might not biomechanically result in increased humeral rotation, possibly because the load on the ISP is compensated by the SCP.

## Materials and methods

### Specimen preparation

**Fig. 4:**
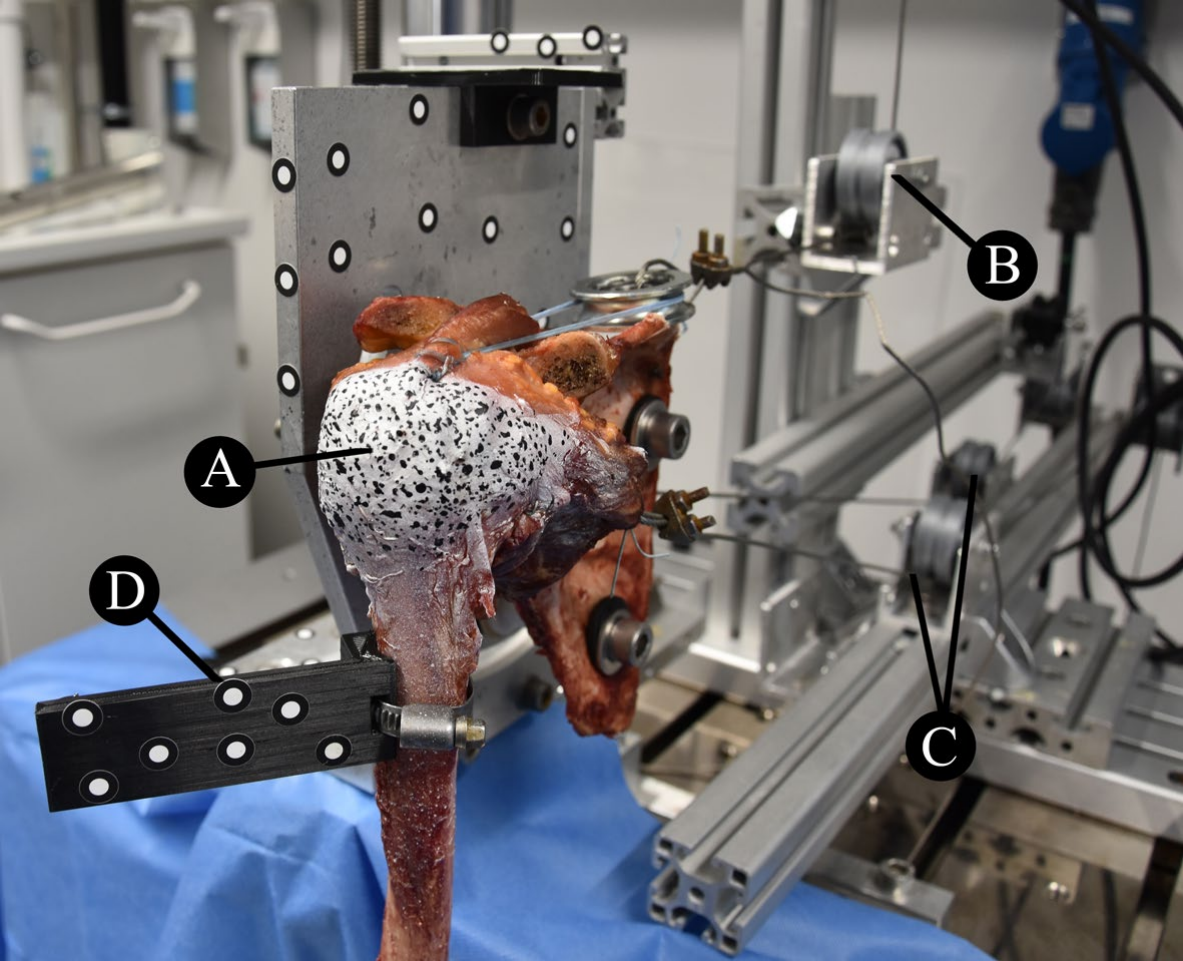
Specimen mounted in testing machine. **A** Stochastic pattern speckled onto surface of rotator cuff muscles (supraspinatus and infraspinatus) for digital image correlation strain analysis. **B** Supraspinatus tendon connected to material testing machine via a cable–pulley system. **C** Infraspinatus–teres minor and subscapularis tendons constantly loaded through their physiological lines of action via a cable–pulley system. **D** Optical tracking markers fixed to humerus and scapula for 3-dimensional motion measurement

Twelve (*n* = 12) fresh-frozen cadaveric shoulders (mean age, 72 ± 13 years; 5 female; 7 male), without visible rotator cuff damage, were tested in this biomechanical study. The skin and subcutaneous tissue were removed to retain only the humerus, scapula, rotator cuff muscles and joint capsule. Fiducials were placed in the bony landmarks recommended by the International Society of Biomechanics (ISB) for the scapula (root of the spine, inferior angle, and acromial angle) and humerus (most caudal points on lateral and medial epicondyles) [25]. True anterior–posterior (AP) radiographs (Veradius Unit, Philips, Amsterdam, The Netherlands) and computed tomography scans (SOMATON Definition Edge, Siemens, Munich, Germany) were taken prior to testing. The specimens were further prepared as described in a previous study: the distal tendinous insertions of the SSP, subscapularis (SCP), infraspinatus (ISP) and teres minor were sutured with a running locking stitch; the scapula was drilled in three points to allow proper fixation in the testing setup; and an intramedullary rod with an attached weight (1 kg) was cemented into the humeral shaft [19]. A fine speckled pattern was created on the bursal side of both SSP and ISP tendons (Fig. 4A) by uniformly spraying white paint (background) and airbrushing with black plaint (MOTIP DUPLI GmbH, Haßmersheim, Germany).

### Experimental protocol

**Fig. 5:**
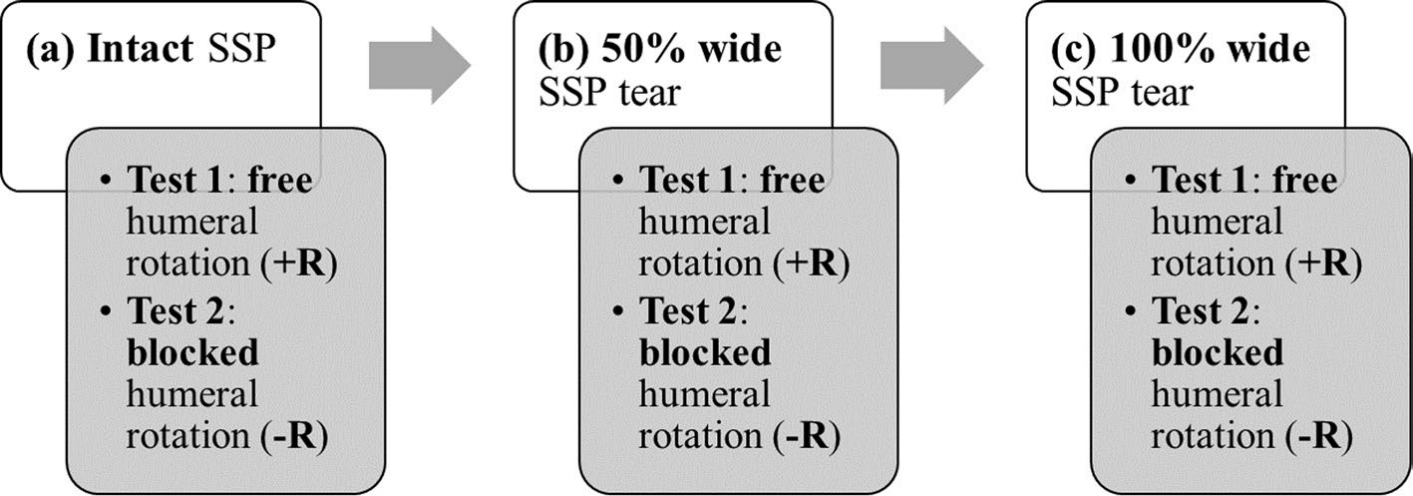
Experimental protocol used to test each specimen. SSP, supraspinatus

The specimens were positioned in the scapular plane and the sutures were secured to wire cables aligned with each muscle’s line of action via a pulley system (Fig. 4B and C). Each muscle's physiological line of action was oriented based on the specimen’s bony landmarks: the line approximately connecting the muscle’s origin midpoint to its insertion midpoint [5, 19]. Optical tracking markers were fixed to the humerus and scapula (Fig. 4D), and the fiducials were digitized using a stereo camera system (ARAMIS 3D Camera 2.3 M, MV550, GOM GmbH, Braunschweig, Germany) with a resolution of 1936 × 1216 pixels. Both SSP and ISP strains and shoulder motion were measured at 10 Hz by the optical tracking system during testing. Glenohumeral abduction until 30° was simulated in the scapular plane by loading the SSP tendon at 2 mm/s (10 kN load cell, EletroPuls E10000, Instron, Norwood, MA, USA), while a constant load was applied to the SCP (15 N) and ISP/teres minor muscles (15 N), in accordance with previous studies [5, 6, 16, 19]. This motion was performed with (1) free humeral rotation (+R) and (2) blocked humeral rotation (−R); and applied to (a) the intact SSP, (b) after a 50% wide (AP) full-thickness tear was created, and (c) after extending the tear to 100% of the AP width of the tendon (Fig. 5). Glenohumeral abduction was performed twice for each test series to increase measurement accuracy and account for variability in the measurements. To restrict abduction to the scapular plane, the humeral rod slid along a low-friction gliding system (igus® GmbH, Cologne, Germany). A metal pin was placed through the rod to block it from rotating, thus constraining humeral rotation. The specimens were divided into two groups based on SSP tear shape: CS tear (*n* = 6) and rLS tear (*n* = 6). Each group was randomly assigned an equal number of right (*n* = 4) and left (*n* = 2) shoulders. The tear was surgically created by detaching a portion of the SSP tendon off its humeral footprint using a scalpel. The AP width of each specimen’s SSP tendon was measured with a caliper prior to testing and taken as the reference for the creation of the 50% and 100% wide tears. The medial–lateral (ML) dimensions of the tears were defined in relation to the measured AP dimensions [19]. In the CS tear group, the tear was created in the posterior SSP footprint, and the ML tear size was defined as 1/4 and 1/2 of the AP SSP dimension for the 50% and 100% wide groups, respectively. In the rLS tear group, the tear was created in the anterior SSP footprint, and the ML tear dimension was equal to the AP SSP size for both the 50% and 100% wide groups.

### Data and image analysis

Each specimen’s scapula and humerus geometries were semi-automatically segmented from the computed tomography scans in 3D Slicer [4] (Version 4.11.20200930 r29402/002be18) and afterwards imported into Aramis Professional (Version 2019, GOM GmbH, Braunschweig, Germany). In order to determine glenohumeral kinematics, bone fixed coordinate systems were created on the humerus and scapula using the anatomical landmarks previously digitized, as recommended by the ISB [25]. The humeral head center of rotation (CoR) was determined by a sphere-fitting algorithm applied to the segmented bone surface. Strain was computed in ARAMIS Professional through digital image correlation analysis. For each test series, glenohumeral abduction was performed twice, and the average of both measurements was calculated at each time point for all force, strain and kinematics data using MATLAB R2018b (MathWorks, MA, USA). Strain was evaluated at the SSP and ISP footprints, as well as at the anterior and posterior borders of each tear, where higher stress concentrations have been observed in SSP tears [11, 18, 19]. To analyze strain magnitude in relation to the range of motion, strain was averaged in 5° increments of abduction, including only specimens that achieved a minimum of 15° of abduction. Maximum SSP and ISP strains were assessed across the full range of motion reached by each specimen, up to the maximum of 30° of simulated abduction.

### Statistical analysis

The effect of constrained humeral rotation on SSP loading, glenohumeral kinematics, and SSP and ISP surface strains was evaluated with a random intercept model in SPSS Statistics (Version 26, IBM, USA). Humeral rotation (free or blocked), tear shape (CS and rLS), and size (50% and 100% wide) were considered as fixed effects, while the specimens were taken as random effects. Significance was set at *P* ≤ 0.05.

## Data Availability

No datasets were generated or analysed during the current study.
